# The Discovery, Characterization, and Quantification of Bioactive Peptides Contained in Palbio Porcine Intestinal Mucosa Hydrolysate Products

**DOI:** 10.3390/ijms26146656

**Published:** 2025-07-11

**Authors:** Sergi Segarra, Carolina de la Torre, Joan Josep Bech-Serra, Bernat Cucurull, Anna Marazuela-Duque, Alejandro Vaquero, Daniel Martínez-Puig, Javier Velasco-Alvarez

**Affiliations:** 1 R&D Bioiberica S.A.U., 08950 Barcelona, Spain; dmartinez@bioiberica.com (D.M.-P.); jvelasco@bioiberica.com (J.V.-A.); 2 Proteomics Unit, Josep Carreras Leukaemia Research Institute, 08916 Barcelona, Spain; carolina.delatorre@gmail.com (C.d.l.T.); jbech@carrerasresearch.org (J.J.B.-S.); bcucurull@carrerasresearch.org (B.C.); 3 Chromatin Biology Laboratory, Josep Carreras Leukaemia Research Institute, 08916 Badalona, Spain; amarazuela@carrerasresearch.org (A.M.-D.); avaquero@carrerasresearch.org (A.V.)

**Keywords:** bioactive peptides, antimicrobial peptides, porcine intestinal mucosa hydrolysate, porcine digestible peptides, animal by-products, peptide quantification

## Abstract

Porcine intestinal mucosa hydrolysates (PIMHs) are by-products of heparin production obtained through a specific enzymatic hydrolysis process, which can theoretically generate bioactive peptides (BAPs). This study aimed to identify, characterize, and quantify BAPs in two Palbio products manufactured by Bioiberica S.A.U. (Palafolls, Spain), which are PIMH protein sources used for animal feed: Palbio^®^ HP (PHP) and Palbio^®^ 62 SP^®^ (P62). Using mass spectrometry (MS)-based peptidomics, we analyzed three samples from each product, fractionated based on molecular weight (<3 kDa, 3 to 10 kDa, and >10 kDa). The <3 kDa fraction was analyzed directly, while the other two fractions were enzymatically digested before MS analysis. The workflow identified 961 peptides in PHP and 1134 in P62. Subsequent bioinformatic analysis using public databases (APD2, StraPep, AHTPDB, and BIOPEP-UWM) led to the identification of six significant BAPs in both PHP and P62, with respective quantified amounts (pg peptide/μg sample): DAVEDLESVGK (0.1626, 0.1939), EGIPPDQQRLIFAGK (0.2637, 0.1852), TITLEVEPSDTIENVK (0.3594, 0.4327), TNVPRASVPDGFLS (1.4596, 0.1898), TNVPRASVPDGFLSEL (8.0500, 0.9224), and VHVVPDQLMAF (0.0310, 0.0054). The first three BAPs are related to antimicrobial activity, while the latter three are associated with cytokine/growth factor-like, antioxidant, and immunomodulatory activities. These bioactivities align with previously reported in vivo benefits observed in animal nutrition using Palbio products. Our findings demonstrate that PHP and P62 are valuable sources of BAPs, supporting their potential role in improving animal health and performance.

## 1. Introduction

Bioactive peptides (BAPs) are specific protein fragments that, in addition to their nutritional properties, have beneficial physiological effects on health [1,2,3,4]. These peptides are typically generated through enzymatic hydrolysis of whole proteins and can come from different sources, including dietary proteins, which release them upon gastrointestinal breakdown [5,6,7].

The use of animal by-products as a protein source in animal nutrition is successfully carried out without adverse effects on the animals. Interestingly, the enzymatic hydrolysis of animal by-products generates peptides that may not only have nutritional value but also provide beneficial effects such as antimicrobial, antioxidant, antihypertensive, or immunomodulatory activities [3,4,6,7,8,9,10]. A specific example of animal by-products used as a protein source in animal nutrition would be porcine intestinal mucosa hydrolysates (PIMHs), also known as porcine digestible peptides (PDPs), which are obtained as by-products of the heparin manufacturing process [11,12] following a circular economy approach. These sustainable protein sources are the result of the valorization of protein-rich liquids that are produced during the production of heparin, and which would otherwise have to be destroyed, resulting in an undesired environmental impact. PIMHs can have different applications aimed at improving human, animal, or plant health. Within the animal nutrition area, three Palbio PIMH products, manufactured by Bioiberica S.A.U. (Palafolls, Spain), are used as highly digestible and palatable protein sources: Palbio HP^®^ (PHP), Palbio 62 SP^®^ (P62), and Palbio 50 RD^®^ (P50) [13]. Soybean meal serves as a carrier in the manufacturing process of P50. These three PIMH Palbio products have been widely utilized for many years in livestock, pet food, and aquaculture nutrition as a source of functional protein in several animal species, including pigs, chicken, calves, dogs, and salmon, providing benefits for their health and well-being (Table 1). However, the bioactive peptide composition of these products remains largely uncharacterized.

Besides these in vivo observations, and in order to better understand the nature and mechanism of action of these products, several in vitro studies have also been performed. More specifically, PIMH P62 demonstrated an in vitro stimulatory effect on the proliferation of skin, liver, intestinal, and lung cell lines, especially when using a fraction with a molecular weight below 200 Da [30]. Other tests support the suitability of Palbio PMIH products to be used in salmon and swine because Palbio’s amino acid profile is comparable to that of these animal species and aligns with their nutritional requirements, as opposed to protein from vegetable sources [31].

Some of these beneficial effects of PIMH Palbio products might possibly be explained by the BAP content of such products. In order to validate this hypothesis, our objective was to identify any potential BAPs in PHP and P62, quantify them, and correlate their associated biological activities with observed in vivo benefits. PIMH P50 was not included in the analyses, given that some BAPs that might be present in the product could come from soybean meal. Soy-derived BAPs have already been studied and recognized for their various potential health applications [32].

## 2. Results

### 2.1. Peptide Identification, Quantification, and Biological Activity

The characterization of the BAPs contained in Palbio products was performed using three samples belonging to different batches of PHP (20/0352, 20/0360, and 20/0361) and three samples from different batches of P62 (19/0044, 20/0006, and 20/0010).

By following a peptidomics and proteomics approach, a total of 961 peptides were identified in PHP and 1134 in P62. A complete list of these peptides is provided in Supplementary Table S1. Notably, many of the peptides were found in the <3 kDa fractions and were analyzed directly without digestion, confirming the presence of naturally occurring small bioactive peptides derived from the original enzymatic hydrolysis process.

**Figure 1:**
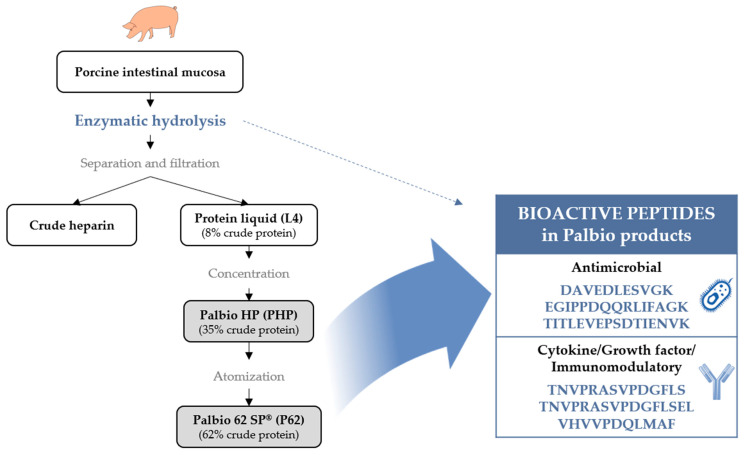
Flow diagram showing the manufacturing process of different porcine intestinal mucosa protein hydrolysates, Palbio products, Palbio HP^®^, and Palbio 62 SP^®^, as well as the generation of bioactive peptides through an enzymatic hydrolysis process.

Table 2 depicts the BAPs that were identified in PHP and P62 after checking public databases, together with their associated biological activity, proteins to which the peptides belong, and amounts. The same six BAPs were identified in both PHP and P62 (Figure 1).

### 2.2. Bioactivity Analysis of Selected Peptides Using BIOPEP ANALYSIS

Functional prediction using the BIOPEP-UWM ANALYSIS CALCULATIONS tool (https://biochemia.uwm.edu.pl/biopep-uwm/, accessed on 23 May 2025) revealed potent inhibitory motifs for ACE and DPP-IV, with particularly strong scores for the peptides TNVPRASVPDGFLS, EGIPPDQQRLIFAGK, and VHVVPDQLMAF, which demonstrated high theoretical potential for cardiovascular and metabolic regulation.

According to the BIOPEP-UWM database [33,34,35], the bioactivity analysis of selected peptides revealed the following bioactive frequencies (BFs):

#### 2.2.1. TNVPRASVPDGFLS

Angiotensin converting enzyme (ACE) inhibitor (BF = 0.4286): potential role in blood pressure regulation and cardiovascular protection; dipeptidyl peptidase (DPP)-IV inhibitor (BF = 0.6429): possible application in diabetes management by enhancing insulin regulation.

#### 2.2.2. TNVPRASVPDGFLSEL

ACE inhibitor (BF = 0.3750): slightly lower activity compared to TNVPRASVPDGFLS but still relevant in cardiovascular applications; DPP-IV inhibitor (BF = 0.5625): potential for glucose metabolism regulation.

#### 2.2.3. DAVEDLESVGK

ACE inhibitor (BF = 0.5455): potential usefulness in regulating blood pressure and cardiovascular health; DPP-IV inhibitor (BF = 0.4545): possible role in metabolic regulation and diabetes management.

#### 2.2.4. EGIPPDQQRLIFAGK

DPP-IV inhibitor (BF = 0.6667): high potential inhibitory effect on DPP-IV, indicating potential benefits in metabolic regulation and diabetes control; ACE inhibitor (BF = 0.6000): high potential for cardiovascular protection through ACE inhibition.

#### 2.2.5. TITLEVEPSDTIENVK

DPP-IV inhibitor (BF = 0.5625): involvement in metabolic regulation and glucose homeostasis; ACE inhibitor (BF = 0.2500): moderate potential in blood pressure regulation and cardiovascular applications.

#### 2.2.6. VHVVPDQLMAF

DPP-IV inhibitor (BF = 0.8182): the strongest DPP-IV inhibition among the analyzed peptides, suggesting a strong role in glucose metabolism and diabetes control; ACE inhibitor (BF = 0.1818): low potential in cardiovascular regulation.

### 2.3. Prediction of Antimicrobial, Antiviral, and Antioxidant Activities Using a Random Forest-Based Machine Learning Algorithm

Given the limited number of experimentally validated peptides with known biological activities, largely due to the lack of studies specifically designed to test them, we implemented three different machine learning algorithms to predict antimicrobial (against Gram-negative and Gram-positive organisms), antiviral, and antioxidant activities.

After training, the performance of the three models was evaluated using a validation dataset. The antimicrobial predictor achieved accuracy, sensitivity, and specificity values of 91%, 89%, and 92%, respectively. The antiviral predictor reached 82%, 85%, and 79%, while the antioxidant predictor obtained 86% for all three performance metrics. Comprehensive lists of all peptides from PHP and P62 and their predicted activities, along with the associated probabilities, are provided in Supplementary Tables S2–S7.

## 3. Discussion

There is currently a growing interest in the use of BAPs in animal nutrition [7]. BAPs found in animal by-products, including PIMHs, may have different biological actions, including antimicrobial, antithrombotic, antioxidant, antihypertensive, cytomodulatory, or immunomodulatory activities, or enhancement of mineral absorption [1,36,37]. This depends on their amino acid class, net charge, secondary structures, sequence, and molecular mass. These activities allow some specific protein sources from animal by-products to enhance feed efficiency and growth performance, along with other health benefits, when used in feeds for livestock or aquaculture species, or even in companion animals [3,7,38].

Data from our studies herein unveiled several antimicrobial and immunomodulatory/growth factor-like BAPs in the Palbio PIMH products PHP and P62. The functionality that was detected is consistent with the origin of the raw material, the production process, and the previous in vivo experimental data obtained with the products. These products come from porcine intestinal mucosa and are generated as by-products of the heparin manufacturing process, through an enzymatic process. In effect, the production of bioactive peptides by alcalase hydrolysis of proteins from different sources has already been described [37,39], and this enzyme is used in the manufacturing process of heparin [40].

The six newly discovered BAPs were identified in both PHP and P62, suggesting that neither the manufacturing process nor the intermediate steps between these two products compromise their integrity. The main transformation from PHP to P62 involves atomization, and our data indicate that the BAPs remain intact following this procedure, as our analyses show that they are preserved. The difference in the total peptide content between the two products may be due to the analytical method employed, inherent variability in peptide quantification, or, more plausibly, the overall low abundance of these peptides in both products, as indicated by our results. In both cases, the absolute quantities may seem low, but the observed values are within a biologically relevant range and comparable to those reported in the literature for enzymatically hydrolyzed animal protein products, and it is well established that bioactive peptides—especially those with immunomodulatory or antimicrobial potential—can exert physiological effects at nanomolar or even picomolar concentrations [41,42].

The wide application of growth promoters and antimicrobials globally over the years in animal production has led to negative consequences [43]. It reduces the sustainability of diets and leads to the accumulation of residues in the environment, causing water and soil pollution as well as in animal products, such as meat, milk, and eggs. The accumulation of residues in products derived from treated animals represents a risk to consumers. Decreasing the use of such products is a key objective in order to reduce the development of antibiotic resistance and allergies [44,45]. It is therefore considered a suitable approach to try and use alternative tools in order to ensure the optimal growth of animals while avoiding the adverse effects of such products. The use of immunomodulatory ingredients in livestock animals has been suggested, and their use is encouraged in order to reduce inflammation and enhance the immune response [46]. The BAPs TNVPRASVPDGFLS, TNVPRASVPDGFLSEL, and VHVVPDQLMAF are part of the sequence of macrophage migration inhibitory factor (MIF; UniProt ID: P14174), an immunomodulatory cytokine involved in immune regulation and inflammatory responses. Some activities previously reported for immunomodulatory BAPs include the enhancement of immune cell functions, seen as a positive impact on lymphocyte proliferation and natural killer cell activity, antibody synthesis, and cytokine regulation [41]. While high levels of MIF have been associated in humans with inflammatory diseases, some studies have identified potential health benefits linked to its expression. In myocardial ischemia, MIF could enhance cardiac health protection by activating AMP-activated protein kinase (AMPK) [47]. In addition, as an integral component of the host antimicrobial response, MIF contributes to the host’s defense mechanisms against infections [48].

The way antibiotics have been used, and often overused, in animal nutrition over the past 50 years has led to the emergence of bacteria resistant to antibiotics and drug residues in meat products. This poses several risks not only for livestock but also for people due to many associated public health implications. Antimicrobial peptides could serve as an alternative to antibiotics and hence help fight antimicrobial resistance, which has become one of the biggest challenges to the human health system. Antimicrobial BAPs of animal origin have shown activity against different Gram-positive and Gram-negative bacteria, as well as against yeast and fungi, which would result in highly convenient applications for animal nutrition [41,43,49,50,51]. These peptides have different mechanisms of action as compared to conventional antibiotics [6]. The characterization of BAPs contained in Palbio products enabled the discovery of three antimicrobial peptides, namely DAVEDLESVGK, EGIPPDQQRLIFAGK, and TITLEVEPSDTIENVK. The first sequence is part of a larger peptide listed in the StraPep database as an antimicrobial peptide (Sequence ID: BP0567). This peptide is derived from dermcidin (UniProt ID: P81605), a protein reported to exhibit antimicrobial activity during the early stages of bacterial colonization [52,53]. The other two peptides belong to a larger sequence (Peptide ID: satpdb12186) described in the APD2 database. This sequence, in turn, is a fragment of ubiquitin (UniProt ID: P0CG47), a protein known for its broad antimicrobial activity against both Gram-positive and Gram-negative bacteria. Additionally, certain fragments of ubiquitin have been reported to possess antifungal properties [54,55].

Our findings support the hypothesis that the health benefits previously observed in animals fed with Palbio products, including improved gut integrity, immune modulation, and reduced pathogen colonization, may be at least partly attributed to their BAP content. This evidence could explain why using Palbio PIMH products as a protein source translates into improved performance and overall health, as observed previously in swine [14,16,18,19,20], poultry [13,23,24,25,26], and aquatic species [29]. It could also explain the enhancement effect on in vitro cell proliferation [30]. Specifically, the presence of immunomodulatory peptides derived from MIF may contribute to enhanced innate immune responses and cytokine regulation, consistent with published data on post-weaning piglets and broiler chickens. These findings could help guide future studies and applications of such products, focusing on the in vivo benefits associated with these bioactivities.

Current scientific evidence supports the use of Palbio products across various animal species at inclusion levels typically ranging from approximately 2.5% to 5% [11,19,28]. At these dosages, there is generally no negative economic impact on feed formulation. In fact, in some cases, the incorporation of Palbio products can even lead to cost savings [19,26,29], enhancing the overall efficiency of feed production. Importantly, the identification of native peptides within the <3 kDa fraction emphasizes the industrial relevance of the original enzymatic hydrolysis process in generating naturally bioactive components, which may serve as strategic tools to reduce the use of antibiotics or synthetic additives in feed formulations. This aligns with current demands for more sustainable, health-promoting approaches in animal production systems.

To further study the biological relevance of the identified peptides, we also worked on functional bioactivity prediction through a combined in silico analysis using both conventional databases and machine learning. Functional prediction using the BIOPEP-UWM ANALYSIS CALCULATIONS tool revealed potent inhibitory motifs for ACE and DPP-IV, with particularly strong scores for the peptides TNVPRASVPDGFLS, EGIPPDQQRLIFAGK, and VHVVPDQLMAF, which demonstrated high theoretical potential for cardiovascular and metabolic regulation. In parallel, we implemented three machine learning classifiers based on Random Forest algorithms, specifically trained to predict antimicrobial, antiviral, and antioxidant activity. These classifiers confirmed the predicted multifunctionality of several peptides already annotated by database searches. High predictive accuracies were obtained with both products, reinforcing their potential relevance in animal health. Additionally, all the analyzed BAPs exhibited notable potential as ACE inhibitors and DPP-IV inhibitors, with possible applications in cardiovascular and metabolic health. Among them, VHVVPDQLMAF demonstrated the highest DPP-IV inhibitory activity, making it a strong candidate for diabetes management. These results provide an initial computational perspective on the bioactive potential of these peptides, but further experimental validation is required to confirm their therapeutic applications.

Several protein sources are used in animal nutrition. Plant-based proteins, especially from soybeans, contain high levels of anti-nutritional factors such as phytic acid, tannins, and alkaloids [56,57]. The animal origin of Palbio products, together with the aforementioned functionalities, would also make them an adequate alternative to vegetable protein sources. On the other hand, such origin might also raise some concerns related to the biosafety of swine protein sources when used in the feed for the same target animal species. This should, however, not be an issue provided that Palbio products are a by-product of the heparin manufacturing process and therefore follow the highest pharma quality standards. Moreover, the hydrolysis technology used in the process guarantees the biosafety of the product [58].

Antimicrobial and immunomodulatory peptides from natural sources offer natural alternatives to antibiotics in livestock, enhancing gut health, immunity, and productivity [46]. Their integration into feed formulations promotes sustainable and eco-friendly agriculture. In human health, these peptides show promise as novel therapeutics against drug-resistant pathogens. Research should focus on optimizing stability, bioavailability, and mechanisms of action to enhance efficacy. Moreover, advances in encapsulation and controlled-release systems could improve peptide delivery. The continued exploration of these bioactive compounds can lead to innovative solutions benefiting global health and nutrition. Their development has the potential to revolutionize disease prevention, treatment, and nutritional strategies.

The main limitation of these studies is the absence of in vitro testing to verify the biological activities of the BAPs identified in the analyzed samples. Such testing could serve as a foundation for future research aimed at evaluating the effects of Palbio products on specific microorganisms, as well as their influence on growth and immune modulation. Even if our studies identified antimicrobial peptides contained in these products, it would be ideal to confirm this activity experimentally. Nevertheless, there is actually in vivo published data supporting the antimicrobial effects of Palbio products, which are very likely provided by the above-mentioned antimicrobial peptides contained in the product. These effects were reported in a feeding trial performed in piglets evaluating gut microbiota–metabolome responses to dietary supplementation with Palbio [20]. More specifically, this study showed that Palbio administration to piglets modulated the intestinal microbiome and led to significantly decreased relative abundance of amplicon sequence variants (ASVs) of opportunistic pathogens from the Streptococcus genus following microbiota analyses from digesta samples. Antimicrobial usage in pig farming used to be very high but is now decreasing, which is suggested to be an efficient way of fighting antimicrobial resistance [59]. This type of in vivo validation, compared to in vitro studies, represents a more valuable piece of evidence in order to correlate the bioactive peptides contained in Palbio products with their biological activity and actual effects when administered to animals. These findings provide valuable in vivo evidence linking the BAPs contained in Palbio to their biological effects. While preliminary, these data underscore the antimicrobial potential of these compounds. Even so, complementary in vitro studies would be beneficial to further validate and characterize these activities under controlled experimental conditions. Also, considering these findings, further research into these BAPs could be valuable for developing antibiotic-sparing strategies and functional protein sources for animal production.

## 4. Materials and Methods

### 4.1. Sample Preparation and Peptide Extraction

**Figure 2:**
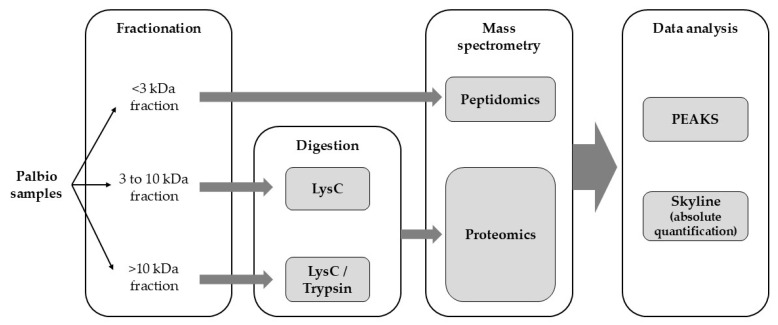
Peptidomics/proteomics workflow description.

Three independent batches of PHP and P62 PIMH products were analyzed using a peptidomics/proteomics approach (Figure 2).

For each batch, approximately 50 mg of powdered sample was weighed and reconstituted in 1 mL of 10% aqueous formic acid (FA). This solution was vigorously vortexed for two minutes to ensure full solubilization of proteins and peptides. The homogenized samples were then subjected to ultrasonic disruption in cold conditions using an ultra-tub sonicator (Bandelin Sonorex, Berlin, Germany) for a total of three minutes. Sonication was performed in cycles of 15 s on and 10 s off, with the samples kept on ice to prevent heat-induced degradation. The samples were subsequently centrifuged at 20,000× *g* for 10 min at 4 °C, and the supernatant was carefully collected into clean microtubes for downstream processing.

### 4.2. Molecular-Weight-Based Fractionation

The soluble peptide-rich supernatants were fractionated according to molecular weight using centrifugal filter units (Amicon Ultra, Millipore, Burlington, MA, USA) with cut-off thresholds of 10 kDa and 3 kDa. This sequential filtration yielded three distinct fractions: peptides smaller than 3 kDa (<3 kDa fraction), peptides between 3 and 10 kDa (3–10 kDa fraction), and proteins or large peptides greater than 10 kDa (>10 kDa fraction). Each fraction was individually collected, dried using a centrifugal vacuum concentrator (SpeedVac, Thermo Scientific, Waltham, MA, USA), and subsequently resuspended in a denaturing buffer consisting of 6 M urea and 0.1 M Tris-HCl, pH 7.0, in preparation for digestion and analysis. Fractionation was necessary to uncover the complete repertoire of bioactive sequences, but since the biological effects of Palbio products derive from the complete peptide matrix, the total peptide yield per fraction is not reported in the main results.

### 4.3. Enzymatic Digestion of Selected Fractions

The <3 kDa fraction, which was expected to contain native peptides produced during the original industrial enzymatic hydrolysis, was analyzed directly using a peptidomics approach without enzymatic digestion. In contrast, the 3–10 kDa and >10 kDa fractions underwent controlled enzymatic digestion to enable proper proteomic analysis. Prior to digestion, the samples were subjected to a reduction step using 10 mM dithiothreitol (DTT) for 30 min at room temperature, followed by alkylation with 55 mM iodoacetamide (IAA) for 30 min in the dark to prevent disulfide bond reformation. The 3–10 kDa fraction was digested overnight (16 h) at 37 °C using 1 µg of Lys-C endoproteinase (Wako Chemicals, Richmond, VA, USA) under mild agitation. The >10 kDa fraction was sequentially digested: first with 1 µg of Lys-C for 3 min and then with 1 µg of sequencing-grade trypsin (Promega, Madison, WI, USA) for 16 min under the same conditions. All digested and undigested peptide fractions were then desalted using C18 reversed-phase ZipTips (Millipore), vacuum-dried, and resuspended in 0.1% trifluoroacetic acid (TFA) prior to LC-MS/MS analysis.

### 4.4. LC-MS/MS Acquisition

Peptide analysis was performed using a nano-liquid chromatography system (Dionex Ultimate 3000, Thermo Scientific) coupled to a high-resolution Orbitrap Fusion Lumos™ Tribrid mass spectrometer (Thermo Scientific) equipped with a TriVersa NanoMate nano-electrospray ionization source (Advion, Ithaca, NY, USA). Chromatographic separation was carried out using a trap column (Acclaim PepMap100, C18, 100 μm × 2 cm, 5 μm, 100 Å) in line with an analytical column (NanoEase MZ HSS T3 C18, 75 μm × 250 mm, 1.8 μm, 100 Å, Waters, Milford, MA, USA). A 90 min linear gradient was employed for peptide elution, consisting of a progression from 3% to 35% buffer B over 60 min, from 35% to 50% in 5 min, and then from 50% to 85% in 2 min. This was followed by a 5 min isocratic elution at 85% B and re-equilibration to initial conditions. Mobile phase A was 0.1% formic acid in water, and mobile phase B was 0.1% formic acid in acetonitrile. The flow rate was maintained at 300 nL/min throughout the run. The mass spectrometer operated in data-dependent acquisition (DDA) mode. Survey MS scans were acquired in the Orbitrap with a resolution of 120,000 at *m*/*z* 200. The most intense precursor ions were selected for fragmentation via higher-energy collisional dissociation (HCD) using a normalized collision energy of 28%. MS/MS spectra were acquired at a resolution of 30,000. Dynamic exclusion was enabled with a 15 s window. The spray voltage was set to 1.60 kV, and the RF lens voltage was tuned to 30%. Singly charged precursor ions were excluded from fragmentation, and positive polarity mode was used throughout.

### 4.5. Peptide Identification and Bioinformatic Annotation

Raw MS data were processed using the PEAKS+ Studio 11 software (Bioinformatics Solutions Inc., Waterloo, ON, Canada), which integrates de novo sequencing results with database searching for improved sensitivity and accuracy. Searches were conducted against the *Sus scrofa* TrEMBL database, which was downloaded in January 2018 and contained 50025 entries. A decoy fusion approach (reversed or randomized sequences) was used to estimate the false discovery rate (FDR). The enzyme was set as “unspecific”, indicating that the cleavage can occur in any amino acid. A minimum de novo sequencing confidence of 15% was set for peptides to be considered in the database search. A peptide-level false discovery rate (FDR) of 5% was applied to all identifications, typically calculated using the target–decoy approach. The minimal and maximum peptide length was set to 4 and 65, respectively. The rest of the parameters were set as follows: precursor mass tolerance (10 ppm), fragment mass tolerance (0.5 Da), fixed modifications (Carbamidomethyl (C)), and variable modifications (Oxidation (M)). The identified sequences were cross-referenced against publicly available databases for known bioactive peptides: APD2 (antimicrobial activity), StraPep (hormones, cytokines, and venom peptides), AHTPDB (antihypertensive activity), and BIOPEP-UWM (antioxidant and enzyme-inhibitory functions).

Afterwards, to promote the bioactive peptide prediction and discovery from all peptides identified (Supplementary Table S1), a machine learning-based algorithm was designed and developed to increase prediction accuracy. In this regard, three different classifiers were implemented using the Random Forest algorithm to predict peptides with antimicrobial, antiviral, and antioxidant activities. The algorithms were trained using a combination of peptides with proven antimicrobial, antiviral, and antioxidant activities, along with peptides lacking known biological activity.

### 4.6. Quantification of Bioactive Peptides

The absolute quantification of selected bioactive peptides was conducted using heavy-isotope-labeled synthetic peptides (AQUA peptides, Thermo Scientific) spiked into the prepared samples. Each AQUA peptide was added in defined amounts (ranging from 0.34 to 1.2 pmol) and co-analyzed with the endogenous peptides under the same LC-MS/MS conditions as described above.

Quantification was performed using Skyline software version 19.1.0.193 by comparing peak areas of light (endogenous) and heavy (spiked-in) peptides. The amounts of peptides injected in the columns are shown in Table 3.

The pg of peptides of Table 3 by μg of the total amount of peptides in the composition was 10, which was calculated according to the following formula:

$$L=\frac{L\;\left(\mathrm{intensity}\;\mathrm{light}\;\mathrm{peptide}\right)}{H\;\left(\mathrm{Intensity}\;\mathrm{heavy}\;\mathrm{peptide}\right)}\times \;H$$

where L/H is the ratio of light/heavy peak areas obtained in Skyline; H is the amount of heavy peptide in pmol injected on column (Sample preparation, Table 2); L is the amount of calculated light peptide in pmol. Final concentrations were expressed as picograms of peptide per microgram of total peptide in the composition.

### 4.7. In Silico Functional Prediction Using BIOPEP-UWM

To assess the potential physiological functions of the identified peptides, the BIOPEP-UWM ANALYSIS CALCULATIONS tool was used [33,34]. This predictive algorithm estimates the frequency and density of bioactive motifs within a peptide sequence, assigning scores for activities such as ACE inhibition, DPP-IV inhibition, immunomodulatory functions, and protease regulation. These in silico predictions complemented both the database-driven functional annotation and the machine learning classifiers, providing a comprehensive evaluation of the bioactivity potential of each identified peptide.

The BF was calculated and provided for each BAP. A BF ≥ 0.5 indicates a high frequency of bioactive motifs. This is considered highly relevant, as more than 50% of the peptide contains known functional sequences. A BF between 0.2 and 0.49 reflects a moderate to good frequency, suggesting interesting functional potential. A BF < 0.2 represents a low density of bioactive fragments and, therefore, the functional effect may be weaker or dependent on enzymatic release and structural factors.

## 5. Conclusions

Palbio PIMH products, PHP and P62, are sources of BAPs with antimicrobial and cytokine/growth factor/immunomodulatory biological activities. These functionalities are consistent with their origin and the preceding enzymatic hydrolysis process. Consequently, it is reasonable to argue that these products might therefore potentially be used for enhancing animal health through dietary supplementation.

## 6. Patents

As a result of the work reported in this manuscript, an application for the grant of a European patent has been made, with application number EP22383164.5.

## Figures and Tables

**Table 1:** Scientific research reporting the in vivo beneficial effects on the health of several animal species of using PIMH Palbio^®^ products as a protein source in their feed.

Animal Species	Main Effects	Reference
Piglets, *Sus scrofa*	Increased average daily feed intake and prevention of intestinal villus atrophy.	Borda et al., 2005 [14]
Piglets, *Sus scrofa*	Improved palatability of postweaning diets, compared to spray-dried porcine plasma.	Martínez-Puig et al., 2007 [15]
Piglets, *Sus scrofa*	Improved piglet post-weaning performance and profitability following partial replacement of spray-dried porcine plasma and soybean protein concentrate.	Solà-Oriol et al., 2010 [16]
Piglets, *Sus scrofa*	Improved palatability in piglets.	Solà-Oriol et al., 2011 [17]
Piglets, *Sus scrofa*	Alternative to sweet milk whey in post-weaning diets without affecting performance.	Figueroa et al., 2016 [18]
Piglets, *Sus scrofa*	Improved intestinal health of pigs after partial replacement of soybean meal and spray-dried plasma.	González-Solé et al., 2020 [11]
Piglets, *Sus scrofa*	Improved performance during post-weaning and grower-finisher phases after use as an alternative to milk and soy protein.	Middelkoop et al., 2023 [19]
Piglets, *Sus scrofa*	Improved gut health in piglets through modulation of their gut microbiota and metabolome.	Segarra et al., 2023 [20]
Broiler chickens, *Gallus gallus domesticus*	Determination of apparent metabolizable energy when used in diets for broilers.	Solà-Oriol et al., 2009 [21]
Broiler chickens, *Gallus gallus domesticus*	Reduced nitrogen excretion and risk of developing enteritis after partial replacement of soybean meal.	Garcés et al., 2010 [22]
Broiler chickens, *Gallus gallus domesticus*	Improved growth performance of broiler chicken, especially from 1 to 21 days of life.	Frikha et al., 2014 [13]
Broiler chickens, *Gallus gallus domesticus*	Improved growth performance in broilers.	Mateos et al., 2014 [23]
Broiler chickens, *Gallus gallus domesticus*	Enhanced performance and profitability after partial replacement of soybean meal.	Salvador et al., 2021 [24]
Broiler chickens, *Gallus gallus domesticus*	Use in non-vegetable-based diets supports recovery from necrotic enteritis.	Jones et al., 2022 [25]
Broiler chickens, *Gallus gallus domesticus*	Beneficial effects on the performance of partially replacing vegetable protein sources in broiler chicken.	Salvador et al., 2022 [26]
Calves, *Bos taurus*	Use as a partial replacement for dried skimmed milk without impairing the performance of calves.	Terré et al., 2009 [27]
Domestic dogs, *Canis familiaris*	Enhanced palatability and digestibility when used as a partial replacement for poultry meal.	Segarra et al., 2023 [28]
Atlantic salmon, *Salmo salar*	Reduction in the diet cost and non-inferiority in terms of performance when used as a partial replacement of fish meal.	Segarra et al., 2022 [29]

**Table 2:** Bioactive peptides found in the samples and associated biological activities, targeted peptides amount expressed in average pg peptide/μg of total amount of peptides, and proteins to which the peptides belong.

Bioactive Peptide	Biological Activity	Protein	Amount in PHP	Amount in P62
TNVPRASVPDGFLS	Cytokine/growth factor (immunomodulation)	Macrophage migration inhibitory factor (UniProt ID: P14174)	1.4596	0.1898
TNVPRASVPDGFLSEL	Cytokine/growth factor (immunomodulation)	Macrophage migration inhibitory factor (UniProt ID: P14174)	8.0500	0.9224
VHVVPDQLMAF	Cytokine/growth factor (immunomodulation)	Macrophage migration inhibitory factor (UniProt ID: P14174)	0.0310	0.0054
DAVEDLESVGK	Antimicrobial	Dermcidin (UniProt ID: P81605)	0.1626	0.1939
EGIPPDQQRLIFAGK	Antimicrobial	Ubiquitin (UniProt ID: P0CG47)	0.2637	0.1852
TITLEVEPSDTIENVK	Antimicrobial	Ubiquitin (UniProt ID: P0CG47)	0.3594	0.4327

**Table 3:** Targeted heavy peptides amount injected into the column.

Peptides	Pmols on Column
TNVPRASVPDGFLS	0.73
TNVPRASVPDGFLSEL	1.20
DAVEDLESVGK	0.55
EGIPPDQQRLIFAGK	0.34
TITLEVEPSDTIENVK	0.47
VHVVPDQLMAF	0.50

## Data Availability

Data are available from the corresponding author upon reasonable request. The mass spectrometry proteomics data have been deposited in the ProteomeXchange Consortium via the PRIDE partner repository with the dataset identifier PXD063531.

## Supplementary Materials

The supporting information can be downloaded at: https://www.mdpi.com/article/10.3390/ijms26146656/s1.
